# LncRNAs downregulated in childhood acute lymphoblastic leukemia modulate apoptosis, cell migration, and DNA damage response

**DOI:** 10.18632/oncotarget.20817

**Published:** 2017-09-11

**Authors:** Romain Gioia, Simon Drouin, Manon Ouimet, Maxime Caron, Pascal St-Onge, Chantal Richer, Daniel Sinnett

**Affiliations:** ^1^ Division of Hematology-Oncology, Research Center, Sainte-Justine University Health Center, Montreal, QC, Canada; ^2^ Department of Pediatrics, Faculty of Medicine, University of Montreal, Montreal, QC, Canada

**Keywords:** long non-coding RNA, acute lymphoblastic leukemia, DNA damage response, treatment resistance, apoptosis

## Abstract

Childhood acute lymphoblastic leukemia (cALL) accounts for 25% of pediatric cancers and is one of the leading causes of disease-related death in children. Although long non-coding RNAs (lncRNAs) have been implicated in cALL etiology, progression, and treatment response, little is known about their exact functional role. We had previously sequenced the whole transcriptome of 56 cALL patients and identified lncRNA transcripts specifically silenced in tumoral cells. Here we investigated the impact of restoring the expression of three of these (*RP11-624C23.1, RP11-203E8*, and *RP11-446E9*) in leukemic cell lines had dramatic impact on cancer hallmark cellular phenotypes such as apoptosis, cell proliferation and migration, and DNA damage response. Interestingly, both *RP11-624C23.1* and *RP11-203E8* had very similar impacts on DNA damage response, specifically displaying lower γ-H2A.X and higher apoptosis levels than control cells in response to genotoxic stress. These results indicate that silencing *RP11-624C23.1* or *RP11-203E8* could provide a selective advantage to leukemic cells by increasing resistance to genotoxic stress, possibly by modulating the DDR pathway. Since genotoxic agents are fundamental parts of antineoplastic treatment, further investigation of the mechanisms these lncRNAs impact would provide novel and interesting avenues for overcoming treatment resistance.

## INTRODUCTION

Childhood acute lymphoblastic leukemia (cALL) is the most frequent cancer in children between 1 and 14 years old and accounts for ≈25% of all pediatric tumours [1]. Precursor B cell cALL (pre-B cALL) is the predominant form of cALL accounting for 85% of cALL patients. Several studies have described expression signatures for classifying molecularly-defined ALL subtypes and improving outcome prediction [2–9]. A new class of molecule, long non-coding RNAs (lncRNAs), play regulatory roles in various processes including pluripotency and tumorigenesis [10, 11]. Recent studies have highlighted their involvement in leukemia initiation and progression. Indeed, the *BALR-2* and *BALR-6* lncRNAs were found to be involved in cell survival or glucocorticoid response in both human and mouse B cells [12, 13]. Furthermore, we and others have demonstrated that lncRNA transcription profiles can discriminate pre-B cALL subtypes accurately [2, 12, 14, 15] or can act as prognostic biomarkers [2]. Although the importance of lncRNAs in tumor biology is clear, little is known about their precise function. Here we studied the impact of three lncRNAs whose transcription is repressed in a cohort of pre-B cALL samples [14, 15]. We found that they significantly affected proliferation, migration, response to cytotoxic drugs, and DNA damage response when overexpressed in pre-B leukemic cells. These findings shed light on lncRNA function in leukemia and point the way towards new biomarkers and therapeutic targets.

## RESULTS

### Overexpression of lncRNAs in pre-B cALL increases apoptosis in response to genotoxic stress

**Figure 1:**
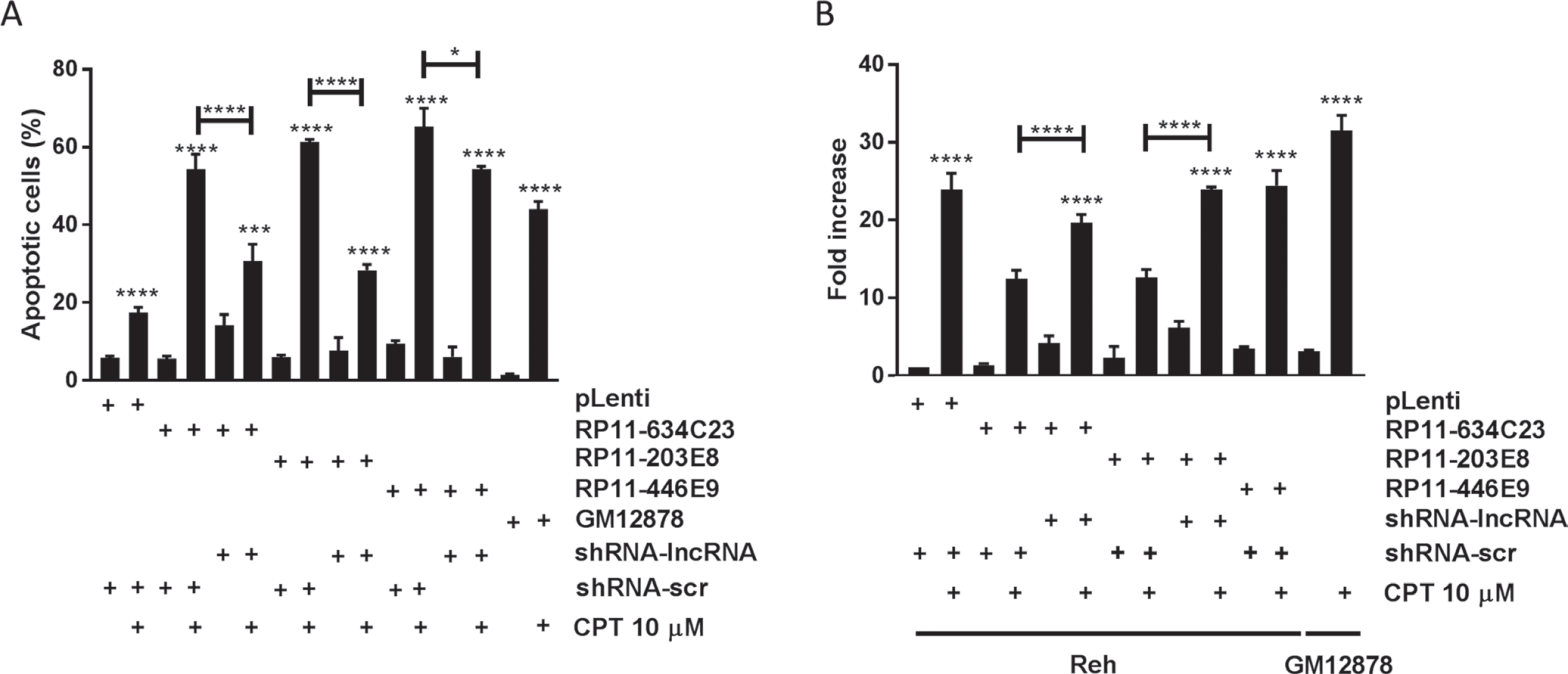
The lncRNAs RP11-624C23.1 and RP11-203E8 are involved in camptothecin resistance and DNA damage response Reh-pLenti, Reh-RP11-624C23.1, Reh-RP11-203E8, Reh-RP11-446E9, Reh-RP11-624C23.1-sh2, Reh-RP11-203E8-sh2, and Reh-RP11-446E9-sh2 were treated with camptothecin (CPT) 10 μM for six hours. Apoptosis (**A**) and γ-H2A.X (**B**) were then measured. *, *** and ****P ≤ 0.05, ≤ 0.001 and ≤ 0.0001, respectively. The GM12878 non-leukemic lymphoid cell line was used as a normal cell control.

We studied the impact of three lncRNAs, *RP11-624C23.1*, *RP11-203E8*, and *RP11-446E9*, which were downregulated in our pre-B cALL cohort (Supplementary Figure 1 and 2 [14, 15]) by overexpressing them in the Reh pre-B cALL cell line (Supplementary Figure 3). We assessed their impact on apoptosis in response to camptothecin (CPT; a DNA-damaging agent), doxorubicine (DOX; an anthracycline), and prednisolone (a corticosteroid). All three lncRNAs significantly increased apoptosis after CPT exposure: > 2-fold increase in *RP11-624C23.1*-overexpressing cells and a > 3-fold increase for those overexpressing *RP11-203E8* or *RP11-446E9* (Figure 1A). However, no effect was observed upon either DOX or prednisolone treatment (Supplementary Figure 6). This increased sensitivity to CPT disappeared in rescue experiments, where cell lines overexpressing these lncRNAs are stably transfected with shRNAs targeting those (Figure 1A and Supplementary Figure 4). The increased sensitivity to CPT upon *RP11-624C23.1*, *RP11-203E8*, or *RP11-446E9* overexpression was also seen in another pre-B cALL cell line, NALM-6 (Supplementary Figure 7), indicating it is not cell-line specific.

### RP11-624C23.1 and RP11-203E8 modulate DNA damage response

The increased sensitivity to CPT prompted us to assess whether these lncRNAs modulate DNA damage response (DDR). To address this question, we quantified phosphorylated histone H2A.X (γ-H2A.X) levels, a well-known DNA damage marker, after CPT treatment. We observed that γ-H2A.X levels decrease to that of control in cells overexpressing *RP11-624C23.1* or *RP11-203E8*, but not *RP11-446E9*, and that γ-H2A.X levels are restored in rescue experiments (Figure 1B). Taken together with the increased sensitivity to CPT, these results suggest that *RP11-624C23.1* and *RP11-203E8* modulate the DDR pathway.

### RP11-446E9 is involved in cell proliferation and migration

**Figure 2:**
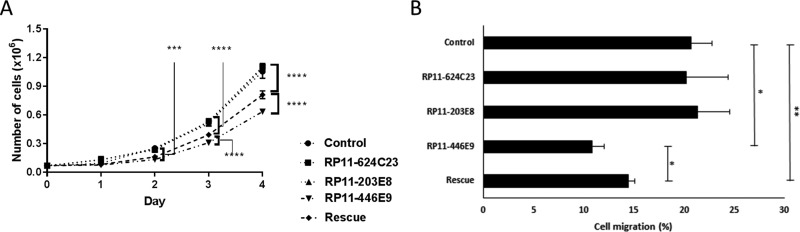
The lncRNA RP11-446E9 regulates cell proliferation and migration Four-day proliferation tests (**A**) and migration assays (**B**) were performed on Reh-pLenti (Control), Reh-RP11-624C23.1 (RP11-624C23), Reh-RP11-203E8 (RP11-203E8), Reh-RP11-446E9 (RP11-446E9), and Reh-RP11-446E9-sh1 (Rescue) cell lines. *, **, *** and ****P ≤ 0.05, ≤ 0.01, ≤ 0.0005 and ≤ 0.0001 respectively.

We observed that cell proliferation was decreased upon *RP11-446E9* overexpression, while it was partially restored (> 50%) in rescue experiments (Figure 2A). Furthermore, transwell migration using SDF1 as chemoattractant led to a *RP11-446E9*-specific decrease in cell migration (Figure 2B). Once again, this effect was reversed in rescue experiments, demonstrating specificity (Figure 2B and Supplementary Figure 4). No effect was seen on cell proliferation or migration for *RP11-624C23.1* or *RP11-203E8* (data not shown).

Taken together, our results indicate that these three lncRNAs play important roles in the regulation of cancer phenotypes such as cell proliferation, migration, and treatment and DNA damage response.

## DISCUSSION

While our knowledge on lncRNAs’ role in cancer has progressed in recent years, only a few have been functionally characterized. Examples of these include *HOTAIR*, which interacts with Polycomb-repressive complex 2 and LSD1 to promote cancer invasiveness [16] and, more recently, *BALR-2*, which is implicated in prednisolone treatment resistance by inhibiting AP-1 activation [12]. Here we showed that three novel lncRNAs downregulated in pre-B cALL, *RP11-624C23.1, RP11-203E8*, and *RP11-446E9*, modulate DDR and migration.

We have shown that both *RP11-624C23.1* and *RP11-203E8* overexpression increased apoptosis and decreased H2A.X phosphorylation upon CPT treatment. Interestingly, the phenotypes affected and the magnitudes of said effects were very similar for both *RP11-624C23.1* and *RP11-203E8* overexpression, possibly indicating that they act in the same molecular pathway. Interestingly, these two lncRNAs flank the *ADAM28* gene, although whether this is related to their function is unclear. However, we see no effect of these lncRNAs overexpression on neighboring genes *ADAM28*, *LYN*, or *RPS* (Supplementary Figure 5), suggesting that these lncRNAs role do not involve neighboring genes’ modulation. These results strongly suggest that these lncRNAs are involved in DDR, possibly acting on DNA damage checkpoints or directly on the DNA repair mechanisms. While further work is required to fully investigate these hypotheses, there are other reports of non-coding RNAs that play roles in DDR and genotoxic stress response [17–20]. These results indicate that silencing *RP11-624C23.1* or *RP11-203E8* could provide a selective advantage to leukemic cells by increasing resistance to genotoxic stress, possibly by modulating the DDR pathway.

While overexpression of *RP11-446E9* also increases apoptosis in response to CPT treatment, it had no impact on H2A.X phosphorylation. This shows that *RP11-446E9* modulates DNA damage-triggered cell death but does not act in the DDR pathway. Furthermore, we have shown that both cell migration and proliferation decrease when *RP11-446E9* expression is restored, suggesting a negative role on cell cycle control and cell motility. More work is required to investigate this thoroughly.

In conclusion, we have functionally characterized three lncRNAs specifically repressed in pre-B cALL: *RP11-624C23.1, RP11-203E8*, and *RP11-446E9*. Restoring their expression in a pre-B cALL cell line promoted tumor suppressor-like phenotypes: apoptosis induction in response to DNA damaging agents and a reduction in cell proliferation and migration. Interestingly, *RP11-624C23.1* and *RP11-203E8* exhibited identical phenotypes, suggesting that they may be involved in a similar pathway. Our results suggest that lncRNAs play key roles in pre-B cALL disease progression and treatment resistance. While it has been shown that lncRNA expression can accurately discriminate pre-B cALL disease subtype and thus be used as a prognostic tool, further work to dissect their exact modes of action is required and could lead to novel, unexplored therapeutic strategies to overcome treatment resistance by targeting the biological pathways associated with their action.

## MATERIALS AND METHODS

### Childhood ALL sample cohort and transcriptome profiling

Our cohort, transcriptome sequencing and analysis were described in Ouimet *et al*. [14]. Our study cohort consisted of 56 pre-B cALL patients (28 females and 28 males) with a mean age at diagnosis of 6.1 ± 3.6 years. All subjects were French-Canadians of European descent diagnosed in the Division of Hematology-Oncology at the Sainte-Justine Hospital (Montreal, Canada) and part of the Quebec childhood ALL cohort (QcALL) [21]. CD10^+^CD19^+^ cells isolated from human cord blood were used as controls. Total RNA was extracted from white blood cell pellets obtained from bone marrow and peripheral blood at diagnosis using the mirVana Isolation kit (Ambion) according to manufacturer's protocol. Following a DNAse treatment to remove possible contamination by genomic DNA, ribosomal RNAs were removed using the RiboMinus Eukaryote kit (Invitrogen). cDNA libraries were prepared using the SOLiD Total RNA-seq kit based on manufacturer's protocol and sequenced on the Life Technologies SOLiD 4/5500 System (paired-end: 50 × 35 bp and 75 × 35 bp). Reads were aligned to the human genome (hg19) using the Lifescope Genomic Analysis Software (Applied Biosystems; Whole Transcriptome Analysis pipeline, default parameters). Expression levels by gene were determined with the HTseq-count software [22]. using gene models from Ensembl75 combined with (non-overlapping) lncRNA transcripts provided in Casero et al. [23]. The identification of differentially expressed transcripts relative to HCB controls was done using the Generalized Linear Model implemented in the edgeR package [24]. The Sainte-Justine Institutional Review Board approved the research protocol, and informed consent was obtained from all participating individuals and/or their parents.

### lncRNA overexpression constructs

cDNA for *RP11-624C23.1*, *RP11-203E8*, and *RP11-446E9* were amplified from CD19^+^ primary cells then cloned into pLenti-CMV-Puro-DEST using the Gateway system (ThermoFisher, Waltham, MA, USA). Short hairpin RNA for *RP11-624C23.1* (5′-AAGGGAUACACUACUGUUAUGGG-3′), *RP11-203E8* (5′-GUCUCAUGUAGGCUGAAUACUUG-3′) and *RP11-446E9* (5′-CUAGCCAGCUGGACUGCA AUUUC-3′) were designed (uptime Life Technologies, Carlsbad, CA, USA) then cloned into psiLVRH1H (GeneCopeia, Rockville, MD, USA). A list of PCR primers used can be found in Supplementary Table 1.

### Cell culture and lentiviral infections

The Reh pre-B cALL and GM12878 B-lymphoblast cell lines (American Type Culture Collection, MD, USA) were cultured in RPMI 1640 (Wisent Bio Products, QC, Canada) supplemented with fetal calf serum (Wisent Bio Products) and 100 units/mL penicillin and streptomycin (Wisent Bio Products) at 37°C in a humidified atmosphere at 5% CO_2_. Cells infected with lentiviral plasmids pLenti-CMV-Puro-DEST or psiLVRH1H were cultured by adding puromycin (1 μg/mL) or hygromycin (400 μg/mL) (Sigma-Aldrich, MI, USA), respectively, to the cell culture medium. 600 ng of the lentiviral particles generated using these constructs were used to infect 1 × 10^6^ cells in 1mL RPMI 1640 supplemented with 10% fetal calf serum and 8 μg/mL polybrene (Sigma-Aldrich). After 24 h, cells are washed with PBS and cultured in 1mL RPMI supplemented with 10% fetal calf serum. Cells expressing the constructs were selected by addition of the appropriate drug, as detailed above.

### Total RNA purification and reverse transcription

The RNeasy Mini Kit (QIAGEN, Toronto, ON, Canada) was used to extract total RNA as per manufacturer's instructions. First-strand cDNA synthesis was done from 1 μg total RNA using the QuantiTect Reverse Transcription Kit (QIAGEN, Mississauga, ON, Canada). QPCR was done using SYBR Green PCR Master Mix (Life technologies). The cycling conditions were as follows: 5 min at 95°C, followed by 35 cycles of 95°C (5 sec), 50°C (30 sec) and 72°C (32 sec). Relative expression levels were normalized to *GAPDH*. QPCR primers used are listed in Supplementary Table 2.

### Cell-based functional assays

#### Apoptosis

Apoptosis was measured using 1 × 10^6^ cells with the FITC AnnexinV/Dead Cell apoptosis kit (ThermoFisher) on a FACS Canto II platform (BD Biosciences, CA, USA) 6 hours after 10 μM camptothecin (Sigma-Aldrich) treatment.

#### Proliferation

Cells were inoculated in triplicate at 0.9 × 10^4^ cells/mL in 150 μL in a 96-well plate and were counted daily over 4 days with a Z1 Coulter particle counter (Beckman-Coulter, QC, Canada).

#### ϒ-H2A.X assessment

Cells were seeded in 6-well plates at 0.5 × 10^6^ cells/mL per well, treated with 10μM camptothecin, and cultured for 6 h. Cells were fixed with 1% formaldehyde for 15 min and permeabilized overnight at −20°C using 95% ethanol. Cells were hydrated 5 min with TBS + 0.05% Tween and incubated at 4°C overnight with anti-phospho-H2A.X(S139) (613402; BioLegend). Cells were washed twice with TBS + 0.05% Tween and incubated 90 min in the dark with Alexa Fluor 488 goat anti-mouse IgG (A11001; ThermoFisher). Finally, cells were washed twice with TBS + 0.05% Tween, incubated 1 h with 50 μg/mL propidium iodide (Life Technologies, ON, Canada) supplemented with 1 μg/mL RNase I, and analyzed by flow cytometry as described above.

#### Migration assay

Polycarbonate filters with a pore size of 8 μm (BD Biosciences, Bedford, MA, USA) were used as previously described [25]. Briefly, 5 × 10^4^ cells were seeded in the upper chamber of the insert and placed in a six-well plate filled with serum-free RPMI-1640 medium supplemented with 30 nM SDF-1 (Sigma-Aldrich) in the lower chamber. After 1 h, cells in the culture medium in the lower chamber were counted with a Z1 Coulter particle counter (Beckman-Coulter).

### Statistical analysis

Statistical analyses were done using GraphPad Prism 5.0 (GraphPad Software, CA, USA). The two-sided Student's *p*-value used to call statistical significance was *p* ≤ 0.05 for all experiments.

## SUPPLEMENTARY MATERIALS FIGURES AND TABLES



## Abbreviations

cALL: childhood acute lymphoblastic leukemia
lncRNA: long non-coding RNA
shRNA: short hairpin RNA
CPT: camptothecin
DDR: DNA damage response
QcALL: Quebec childhood ALL cohort
qPCR: quantitative polymerase chain reaction
