# Supplementary material for: LncRNAs downregulated in childhood acute lymphoblastic leukemia modulate apoptosis, cell migration, and DNA damage response
Source: Oncotarget. 2017 Sep 11;8(46):80645–50. doi: 10.18632/oncotarget.20817 (PMC5655227; doi:10.18632/oncotarget.20817)
Supplement: Supplementary file 1 [file oncotarget-08-80645-s001.pdf]

LncRNAs downregulated in childhood acute lymphoblastic leukemia modulate apoptosis, cell migration, and DNA damage response

SUPPLEMENTARY MATERIALS

Supplementary Table 1: Characteristics of PCR amplimers

RP11-624C23.1	Forward	5′- GGGGACAAGTTTGTACAAAAAAGCAGGCTGAACAAGTGGGGAGAAATCTTA-3′
	Reverse	5′- GGGGACCACTTTGTACAAGAAAGCTGGGTTGTTATGATTCCCATTTGGGTTG-3′
RP11-203E8	Forward	5′-GGGGACAAGTTTGTACAAAAAAGCAGGCTAAAACTTTATCAATTTCTTATATTTTAT-3′
	Reverse	5′- GGGGACCACTTTGTACAAGAAAGCTGGGTCTCAAATTATACCCTCTTTGTAG-3′
RP11-446E9.2	Forward	5′- GGGGACAAGTTTGTACAAAAAAGCAGGCTTCTCGAACTCCTGACCTCAGGC-3′
	Reverse	5′- GGGGACCACTTTGTACAAGAAAGCTGGGTGTGTTTCACTGGTTTTATTTCACA-3′

Supplementary Table 2: Characteristics of qPCR amplimers

RP11-624C23.1	Forward	5′-CTGAGCCCTCTCTCCCAATA-3′
	Reverse	5′-ATCACTGGGCAGGTCAAGAG-3′
RP11-203E8	Forward	5′-CTCCCCAATCCAAGAAACAG-3′
	Reverse	5′-GCCAAAAATCCTCTCCATCA-3′
RP11-446E9.2	Forward	5′-CCCCGTCCTCTCACAAACTA-3′
	Reverse	5′-GACAACTGGCTGTGGAACCT-3′
ADAM28	Forward	5′-AAGAACTCCCTGGGGTGAAG-3′
	Reverse	5′-AAAGCACTGCAATTTTTCCA-3′
LYN	Forward	5′-CAGTACCAAGGTGGCTGTGA-3′
	Reverse	5′GGCCATGTACTCGGTGATG-3′
GAPDH	Forward	5′-TGCACCACCAACTGCTTAGC-3′
	Reverse	5′-GGCATGGACTGTGGTCATGAG-3′

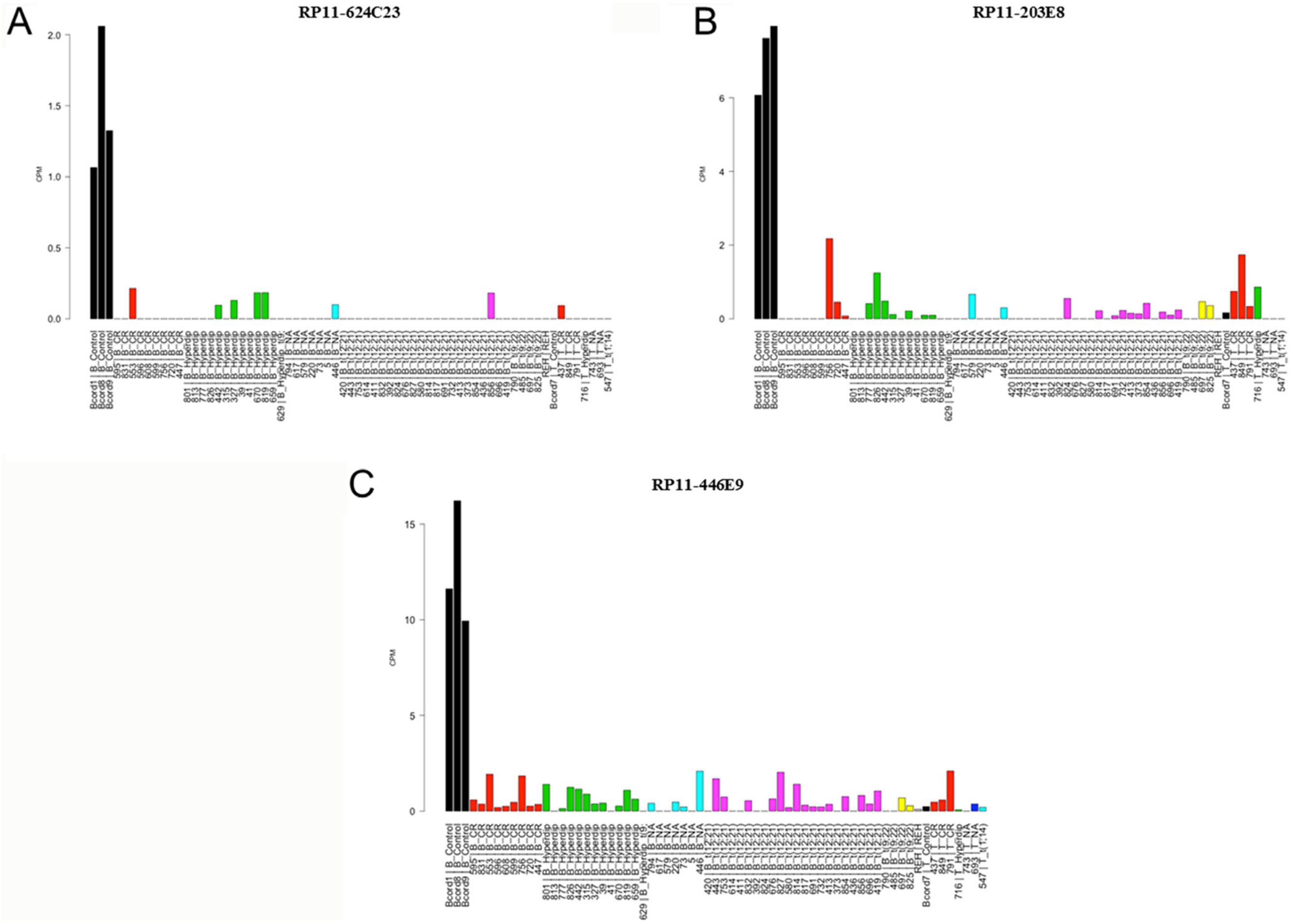

Supplementary Figure 1: The expression of lncRNAs *RP11-624C23*, *RP11-203E8*, and *RP11-446E9* lncRNAs is downregulated in pre-B cALL patients. RNA sequencing was performing on 63 patients, 3 normal samples as control and Reh cell line as described in Ouimet et al. and Lajoie et al.

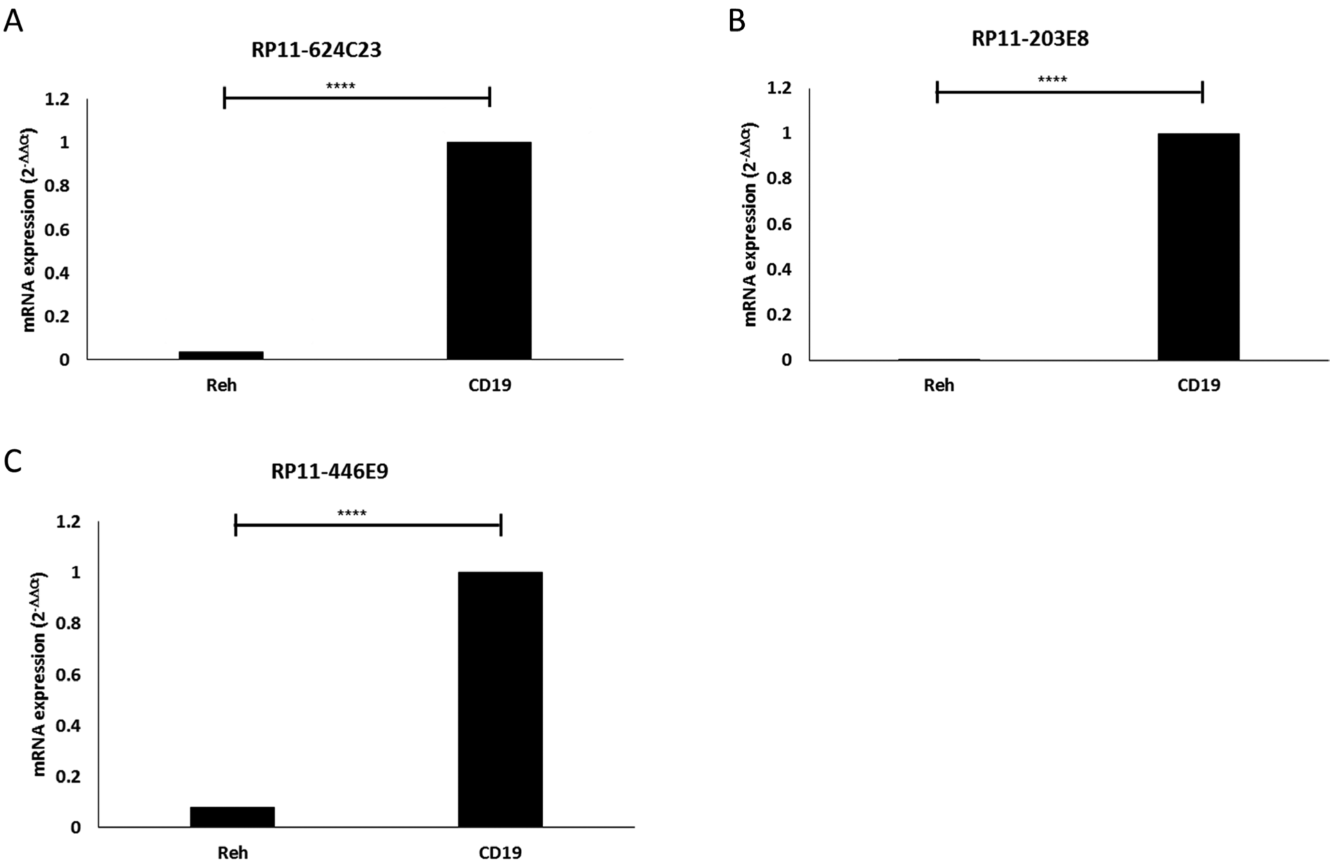

Supplementary Figure 2: LncRNAs *RP11-624C23.1, RP11-203E8,* and *RP11-446E9* are down-regulated in Reh cell line. QPCR of *RP11-624C23.1* (**A**), *RP11-203E8* (**B**), and *RP11-446E9* (**C**) lncRNAs were done on Reh using $CD19^+$ primary cell (CD19) as control. ****$P \leq 0.0001$.

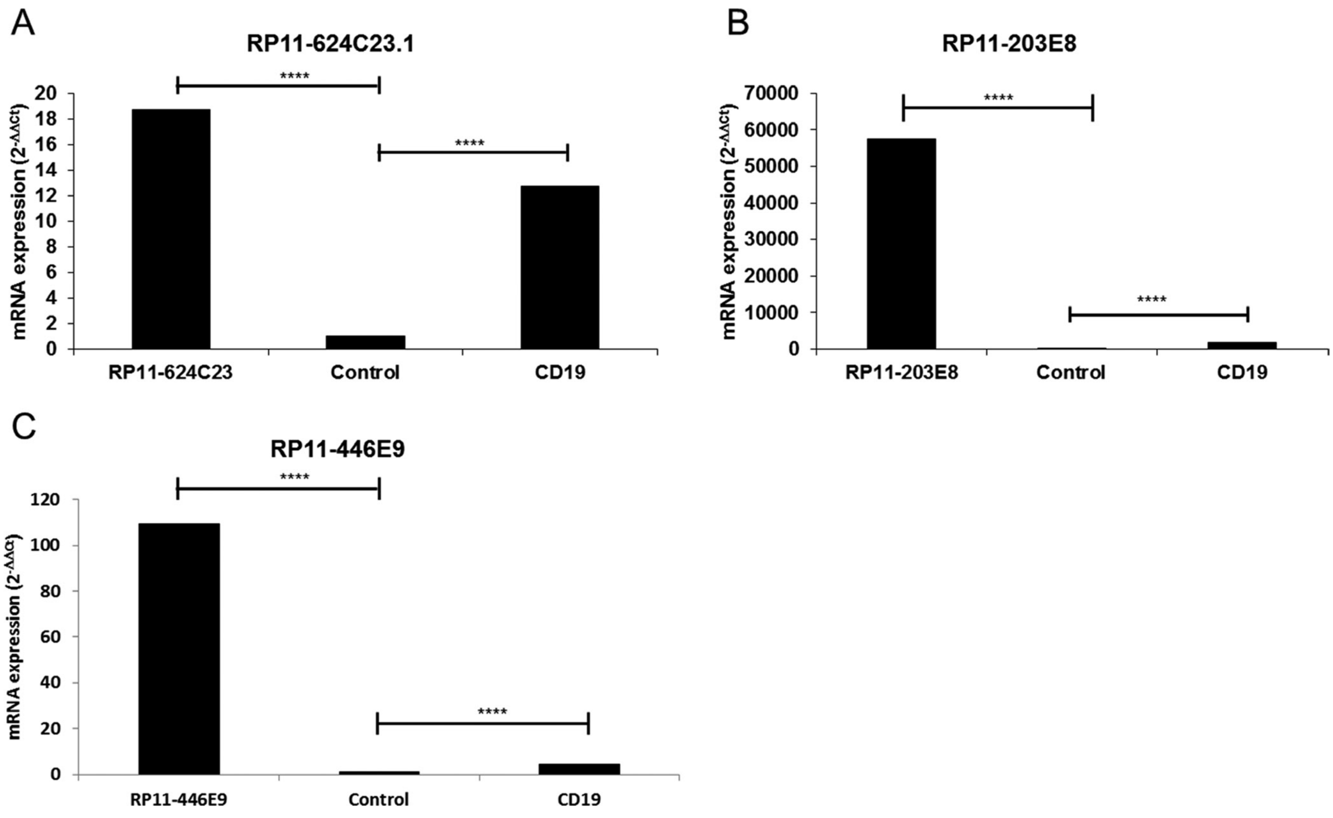

Supplementary Figure 3: LncRNAs *RP11-624C23.1, RP11-203E8,* and *RP11-446E9* are up-regulated in constructs overexpressing them. QPCR of *RP11-624C23.1* (**A**), *RP11-203E8* (**B**), and *RP11-446E9* (**C**) lncRNAs were done on Reh after infection with lentiviral expression vector. $CD19^+$ primary cell (CD19) and Reh-pLenti (Control) were used as controls. Results were normalized on *GAPDH* expression. ****$P \leq 0.0001$.

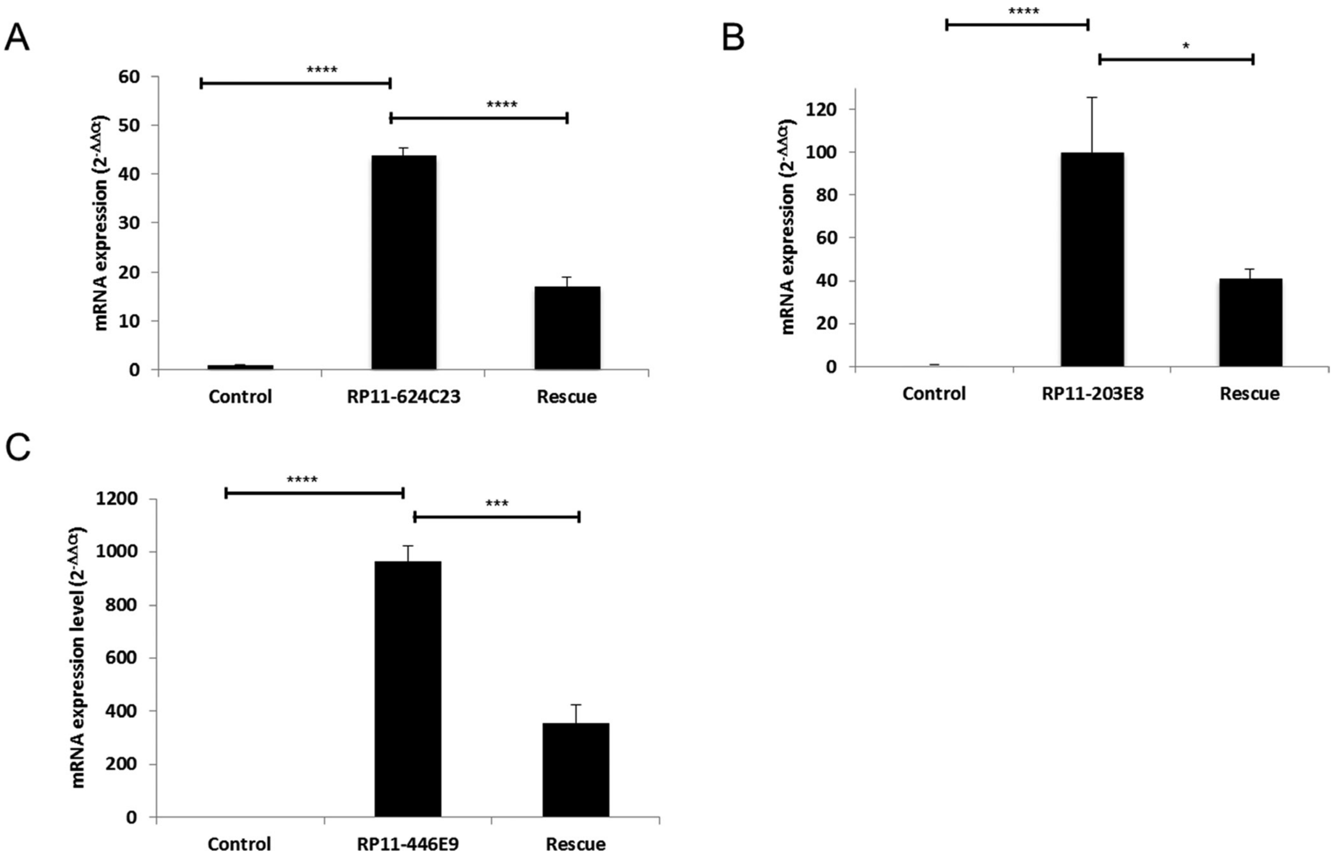

Supplementary Figure 4: Overexpression of *RP11-624C23.1, RP11-203E8,* and *RP11-446E9* is repressed by shRNA-mediated silencing. QPCR of *RP11-624C23.1* (**A**), *RP11-203E8* (**B**) and *RP11-446E9* (**C**) lncRNAs were done on Reh-RP11-624C23.1 (A), Reh-RP11-203E8 (B) and Reh-RP11-446E9 (C) after shRNA-mediated silencing. Results were normalized on *GAPDH* expression. *, *** and ****$P \leq 0.05$, ≤ 0.005 and ≤ 0.0001, respectively.

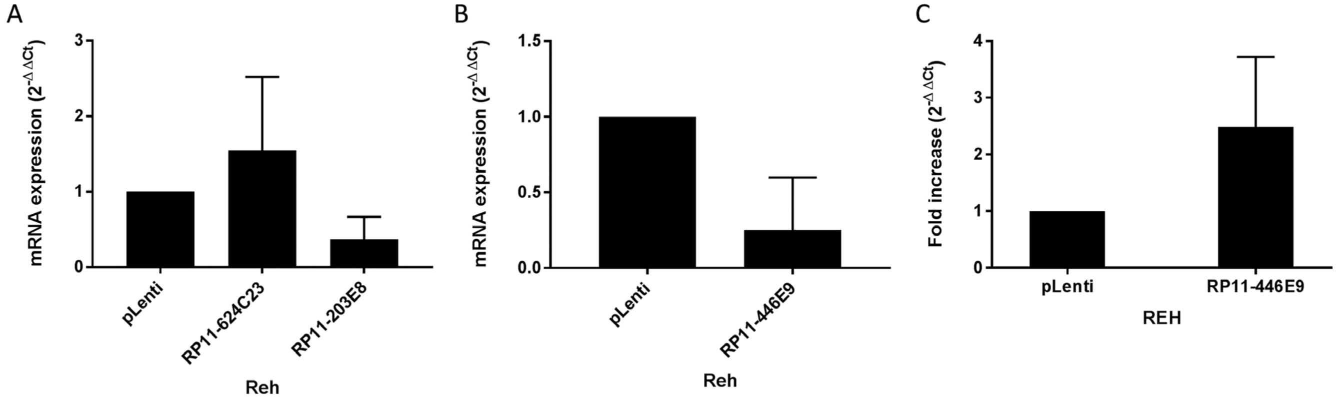

Supplementary Figure 5: Expression levels of genes neighboring *RP11-624C23.1* and *RP11-203E8*. QPCR of *ADAM28* (**A**), *LYN* (**B**), and *RPS* (**C**) were done in control empty vector Reh cells (pLenti) and Reh cells overexpressing *RP11-624C23.1* or *RP11-203E8*. Results were normalized on each respective gene's expression in the pLenti control.

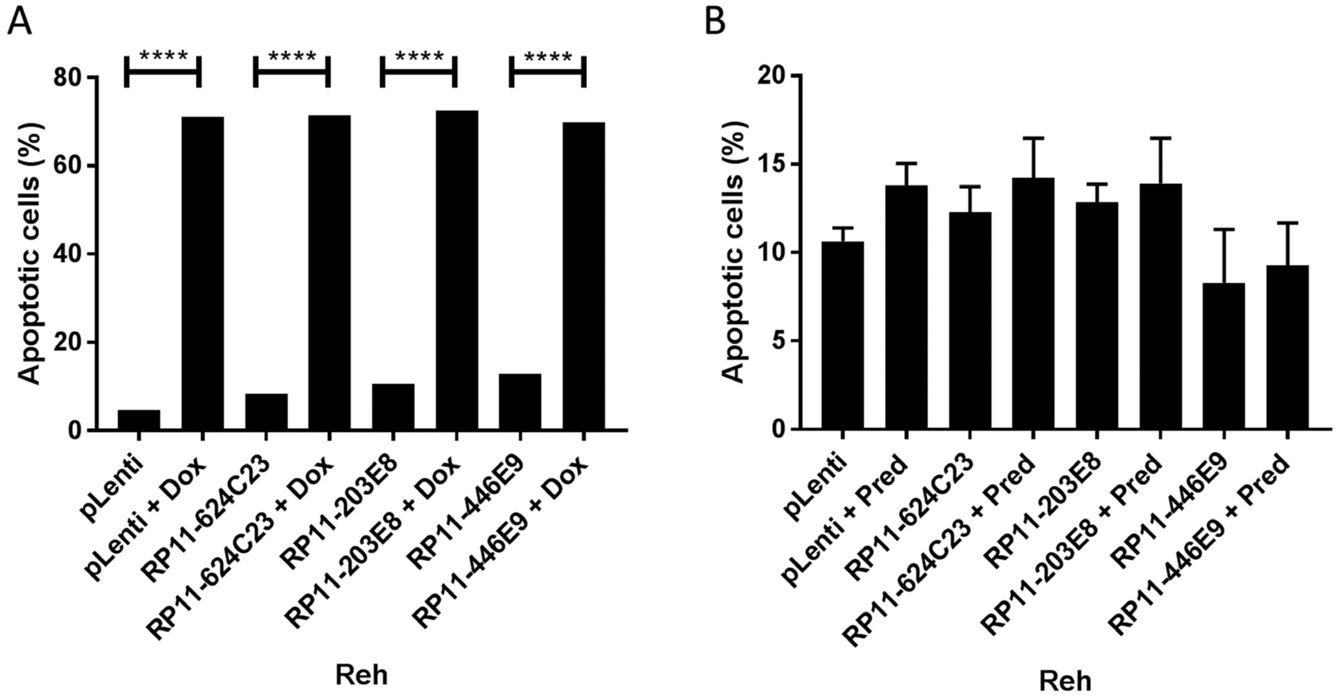

Supplementary Figure 6: Doxorubicine and prednisolone sensitivity are not affected by *RP11-624C23.1, RP11-203E8,* or *RP11-446E9* overexpression. Control (pLenti) Reh cells or Reh cells overexpressing *RP11-624C23.1*, *RP11-203E8*, or *RP11-446E9* were treated with doxorubicine (150 nM, 24 h) (**A**) or prednisolone (750 µM, 24 h) (**B**). Apoptosis was measured using Annexin V/PI.

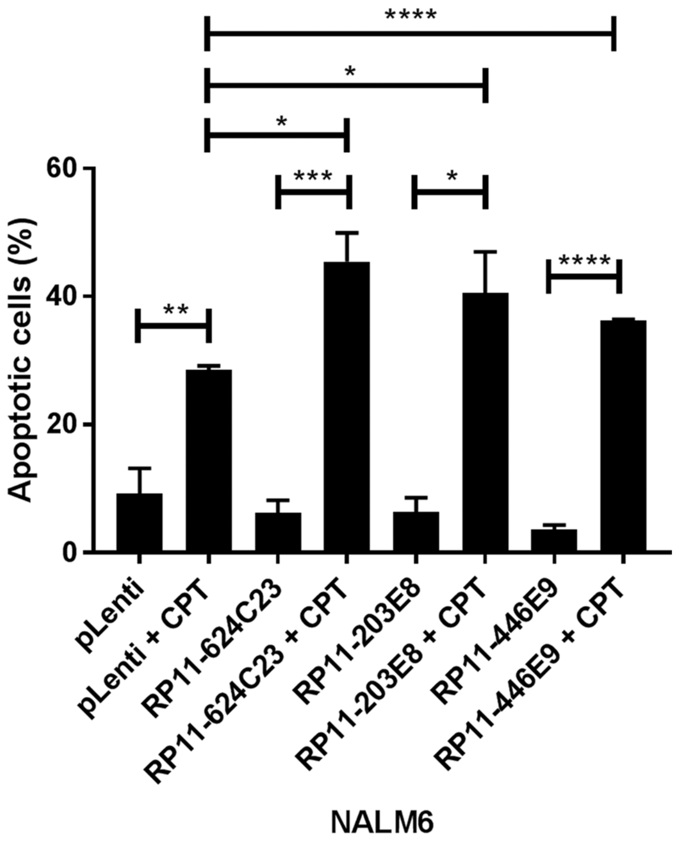

Supplementary Figure 7: NALM-6 pre-B cALL cells overexpressing *RP11-624C23.1, RP11-203E8,* or *RP11-446E9* are sensitive to camptothecin treatment. Control (pLenti) NALM-6 cells or NALM-6 cells overexpressing *RP11-624C23.1*, *RP11-203E8*, or *RP11-446E9* were treated with camptothecin (5 uM, 3 h). Apoptosis was measured using Annexin V/PI.

REFERENCES

1. Ouimet M, Drouin S, Lajoie M, Caron M, St-Onge P, Gioia R, Richer C, Sinnett D. A childhood acute lymphoblastic leukemia-specific lncRNA implicated in prednisolone resistance, cell proliferation, and migration. Oncotarget. 2017; 8:7477–88. https://doi.org/10.18632/oncotarget.13936.

2. Lajoie M, Drouin S, Caron M, St-Onge P, Ouimet M, Gioia R, Lafond MH, Vidal R, Richer C, Oualkacha K, Droit A, Sinnett D. Specific expression of novel long non-coding RNAs in high-hyperdiploid childhood acute lymphoblastic leukemia. PLoS One. 2017; 12:e0174124. https://doi.org/10.1371/journal.pone.0174124.
